# Psychosocial Aspects of Injuries Among Professional Folk Dancers

**DOI:** 10.3390/ijerph22071044

**Published:** 2025-06-30

**Authors:** Csilla Almásy, Anita R. Fedor

**Affiliations:** 1 Faculty of Health Sciences, Doctoral School of Health Sciences, University of Pécs, 4. Vasvári Pál Str., 7622 Pécs, Hungary; 2 Department of Social Sciences and Social Work, Faculty of Health Sciences, Institute of Social Sciences, Quality of Life and Sociology of Health Coordination Research Centre, University of Debrecen, 2-4. Sóstói Str., 4400 Nyíregyháza, Hungary; fedor.anita@etk.unideb.hu

**Keywords:** dancers, injury, psychosocial factors, perceived stress, burnout, coping skills, perceived social support

## Abstract

Injury or fear of injury can cause stress for everyone. This is especially true for dancers, whose careers can be ruined by a serious injury. Stress or various psychological problems can play a role in the development of injury. Our research aims to explore the psychosocial patterns associated with injuries among Hungarian professional folk dancers. A cross-sectional study was carried out with 96 professional dancers (47.9% male, 52.1% female, mean age 29.9 years). Data was collected through an online questionnaire survey. Among psychological factors, perceived stress (using the Perceived Stress Scale), burnout (using the Athletic Burnout Questionnaire), coping skills (using the Athletic Coping Skills Inventory), relationship with the leader (using the Coaching Behaviour Questionnaire) and perceived social support (using the Multidimensional Scale of Perceived Social Support) were examined among injured and non-injured dancers. The two groups were compared along psychological subscales using Multivariate Analysis of Variance (MANOVA) followed by a post hoc ANOVA and Mann–Whitney test regarding social support. Our results showed a significant correlation between psychosocial factors and injuries sustained during the study period. Positive correlation was found between injuries and perceived stress (*p* < 0.001) and burnout (reduced sense of accomplishment *p* = 0.021; dance devaluation *p* < 0.001). Factors reflecting dancer’s behavior and coping skills also correlated with injuries, such as a decrease in coachability (*p* = 0.007), less concern (*p* = 0.029), and negative reactions to the leader’s behavior (*p* = 0.019). In addition, perceived social support from family also negatively correlates with injury (*p* = 0.019). Our findings suggest a multidirectional relationship between physical injuries and the mental state of dancers. Further investigation of the causal relationships is recommended, with the aim of using psychosocial support tools during the prevention and treatment of injuries by the professionals dealing with dance artists. It is also recommended to investigate whether individual psychological factors are directly related to injuries or interact with each other. It would also be useful to introduce prevention programs that help dancers manage their emotions related to injuries.

## 1. Introduction

Professional dancers are often compared to elite athletes due to the high physical and mental demands placed on them [1,2], as they are particularly prone to a wide range of musculoskeletal injuries [3]. In total, 90% of them will experience some kind of injury during their career, and about 50% of them will have persistent or recurrent pain [4,5]. The reason for this exceptionally high incidence is the continuous high workload similar to that of competitive athletes throughout the year, the lack of adequate rest and recovery, and the daily rehearsals and training due to continuous performances [6,7]. Injuries may differ between dance styles [8], but the extensive literature suggests that lower limb (ankle and knee) injuries are the most common among dancers, followed by low back pain [3,9,10]. Hungarian folk dance is rich in skipping, jumping, and leg movements, with powerful footwork, lifting movement, and relatively fast rhythms. Some movements require athletic skills (jumping, leg swings above the head, turns in jumps, landing in a kneeling position from a jump, etc.), so the risk of lower limb and lower back injuries is high among Hungarian folk dancers.

As with athletes, a major injury can compromise or even ruin a dancer’s career [11]. Therefore, special attention needs to be given to the prevention of injuries and the success of injury recovery and return. However, this requires exploring the factors associated with injury. While, in the past, most studies of this kind considered the biomechanical and medical aspects of the injury as well as demographic factors (age), the importance of psychosocial factors is becoming increasingly evident [12,13,14,15]. Athletes play an active role in injury in several ways. Their behavior and actions, or lack thereof, prior to injury can lead to injury. Similarly, their behavior after injury can either support or slow down the recovery process [13]. The fear of reinjury also brings negative consequences for the mental state of athletes and dancers [16]. The reinjury rate can be as high as 20–50% in athletes [17] and around 50% in dancers [5]. As previous injuries are one of the strongest risk factors for injury, whether to the same or nearby body parts [18], the fear of re-injury among athletes and dancers is understandable. The athletes involved reported that they had physically recovered from their injuries but had not been able to cope mentally [17].

We have no information about injuries and associated psychological dysfunctions among Hungarian folk dancers. Therefore, the primary aim of our study is to examine injuries occurring among professional Hungarian folk dancers. Our secondary aim is to compare certain psychological factors between injured and non-injured dancers. To this end, we examine the development of perceived stress, burnout, coping skills, and perceived social support among injured and non-injured dancers.

Our first hypothesis is that lower limb injuries are the most common among Hungarian folk dancers (H1). We also hypothesize that we will find a positive correlation between certain psychological dysfunctions and injuries sustained by dancers during the study period (H2). Finally, we also hypothesize that, with increasing age and thus professional experience, the occurrence of both injuries and psychosocial dysfunction increases (H3).

### 1.1. Stress-Injury Model

Williams and Andersen [19] aimed to develop a stress-injury model to highlight the possible mechanisms linking sports injuries and stress, thereby providing opportunities to reduce stress-related injuries and a framework for further research in this area. The model developed in 1988 was revised by the authors ten years later, taking into account research based on their framework and making refinements [20]. According to the final model, potentially stressful situations (such as strenuous training or a significant competition) can trigger a stress response in athletes, which can result in injury. The stress response manifests itself in two ways: cognitive evaluation of the situation (assessment of demands, personal resources, consequences) and physiological and attentional reactions (muscle tension, narrowing of the field of vision, increased distractibility). The occurrence and magnitude of the stress response are influenced by certain factors: history of stressors (i.e., life events, daily hassles, previous injury), personality (i.e., hardiness, locus of control, sense of coherence, competitive, trait anxiety, achievement motivation, sensation seeking), and coping resources (social support, stress management, other psychological coping skills). These can influence the stress response either independently or in interaction with each other.

The stress-injury model was the first framework to take psychosocial factors into account in the risk of injury in athletes. Since dancers can be considered similar to athletes due to their equally intense physical and mental load, the present study partly uses the stress-injury model as a basis, but our investigation also examined additional psychological factors and their relationship to injury.

### 1.2. Injuries and Perceived Stress

Stress is a psychological and physiological reaction that is perceived as a challenge or threat and is a response to demands or stressors that exceed an individual’s ability to adapt. Stressors can come from external or internal sources [21]. Perceived stress, on the other hand, is defined as how individuals interpret the amount of stress experienced at a given time or time period [22]. In this early concept, the authors highlight the relationship between stress and injuries, describing how negative psychological variables influence an athlete’s behavior in stressful situations (e.g., during a competition, match, or, in the case of dancers, during a performance), both through changes in cognitive function and through altered physical factors that may play a role in an injury. In their consensus statement, Tranaeus et al. [15] confirm that stress response is the strongest psychological risk factor for acute injury. Ivarsson and colleagues [23] reached the same conclusion in their meta-analysis examining the relationship between psychosocial factors and sports injuries.

### 1.3. Injuries and Burnout

The literature is extensive on burnout syndrome (hereafter: burnout). The most widely accepted definition is that of Maslach and Jackson [24], who, based on their empirical research in the workplace, believe that burnout is a multidimensional phenomenon and psychological syndrome that causes emotional exhaustion, depersonalization, and reduced personal performance, and it can occur in people who work with people in some form [24].

Although this definition is indeed widespread, it is limited to the occupational environment. Among athletes—and dancers in our research—it needs to be modified to adjust it to that population. Athletes can experience physical and emotional exhaustion due to strenuous training and competitions. The reduced sense of performance may relate to athletic performance and results achieved. The third dimension, depersonalization, in this context refers to a distancing from sports activities, when sports and results become less important to the athlete. In summary, the three dimensions of burnout in athletes can be modified to physical/emotional exhaustion, sports devaluation, and reduced athletic accomplishment [25]. Dancers experience conditions very similar to those of athletes [2]. Training with high physical and mental workloads for a longer period of time and worries about (re)injury and reduced performance are associated with higher levels of perceived stress in this population [17]. These factors are well-known predictors of burnout in the long term [26,27]. A sense of decreased performance due to injury can lead to a loss of motivation, which, as one of the most significant factors of burnout, can lead to early career termination [28]. In conclusion, the research findings support that athletes and dancers are prone to burnout due to various factors and that burnout is also associated with injuries.

### 1.4. Injuries and Coping Skills

According to the Athletic Coping Skills Inventory [29], an athlete’s coping ability can be examined along three dimensions: coachability, concentration, and worries about performance. These three factors can influence an athlete’s performance.

Coachability can generally be defined as an individual characteristic relating to how they respond to instructions, feedback, and other learning opportunities and how they process them [30]. However, the term originates from sports psychology, where, according to Giacobbi [31], it consists of the following components: the intensity of effort, reactions to coach feedback, openness to learning, trust/respect for the coach, working with teammates, coping with criticism, and is considered an important predictor of athletic success.

Concentration is the sustained maintenance of attention and the filtering out of distractions, which helps individuals focus on the task at hand [32] and increases engagement [33]. In relation to sport, we can define it as meaning that athletes are not easily distracted and are able to focus on the task at hand, both in training and in competition, even in adverse or unexpected situations [34]. The appropriate distribution of attention and better concentration are factors that support performance [35]. These have been linked to higher self-esteem [29] and well-being [36].

Freedom from worry means that athletes are able to keep themselves free from concerns about their performance or mistakes and are not worried about what others think of their performance [34].

According to Williams and Andersen’s stress-injury model [20], athletes with poorer and less effective coping skills are at greater risk of injury. According to the model, one factor in elevated stress response is attentional changes, specifically increased distractibility, which places athletes at greater risk of injury. Devantier [37] also concluded in his study that previous injuries and coping with difficulties are the main predictors of future injuries. A study conducted among dancers by Noh et al. [38] found that freedom from worry is a significant predictor for the frequency and duration of injury.

### 1.5. Injuries and Perceived Social Support

Social support is the combination of resources that an individual receives from others who are important to them such as family, friends, colleagues, etc. [39]. According to one of the earliest definitions, social support means that an individual is loved, cared for, respected, valued, and part of a network that provides mutual assistance [40]. The social environment plays a role in an individual’s well-being, and support from others is seen as a resource. Empirical research has shown, however, that perceived social support, the perception of available help, is more strongly associated with health and mental well-being than actual social support [41,42] and is more significantly responsible for the buffering effects of support [43]. Perceived social support plays a significant role in both athletic performance [44] and psychological health, such as burnout levels of athletes [45,46]. Furthermore, according to the stress-injury model [20], less social support influences the development of injuries through an elevated stress response. Hardy et al. [47] also observed in their research among athletes in several sports that a reduced sense of support is associated with an increase in the frequency of injuries. Patterson et al. [48] conducted a study among dancers and found that perceived social support has a moderating effect and can be a significant predictor of vulnerability. Mainwaring et al. [49] also found in their systematic review that social support is associated with the risk of dance injury.

Interpersonal aspects play a significant role in athletes’ performance. This includes, in particular, cooperation and communication with the coach [50]. Therefore, the literature emphasizes the athletes’ relationship with their coaches and perception of the coach’s behavior as well. The coach’s behavior can influence the athlete’s performance and mental state [41]. A positive coach–athlete relationship promotes participation, athlete satisfaction, self-esteem, and better performance [51]. Relationships with the coach also play a significant role in injuries; however, it remains unclear what impact the coach has on the development of the injury. A poor relationship can be a risk factor for injury [52]; on the other hand, during post-injury rehabilitation, the coach can help the athlete manage the psychological burdens associated with recovery and return to the sport [53]. In his study, Malinauskas [54] concluded that, for athletes, a positive relationship with their coach is more important after an injury than before.

Based on the bio-psychosocial approach to injuries presented above, our research was to investigate the psychosocial changes associated with injuries among Hungarian professional folk dancers.

## 2. Materials and Methods

### 2.1. Sample and Procedure

Between March and June 2024, a cross-sectional study was carried out among Hungarian professional folk dance artists. The ethical approval number for this study is TUKEB BM/819-1/2024. The aim of present research was to examine professional folk dancers. There are currently four professional folk dance companies operating in Hungary. We contacted the leaders in writing and asked the dancers to participate in this research. The selection criteria were as follows: native Hungarian speaker, over 18 years of age, employed as a full-time dancer. Exclusion criteria were as follows: non-native Hungarian speaker, under 18 years of age, not employed as a full-time dancer. During the period under study, approximately 130 professional folk dancers were available, all of whom were recruited either directly or through dance leaders. Ninety-six of them participated voluntarily in this research, resulting in a response rate of 74%. Dancers completed an online questionnaire. The survey covered demographic information, career-related injuries, perceived pain levels, and various psychosocial factors. Respondents were informed that participation in this study was voluntary, that their responses would be anonymous, and that they could discontinue at any time.

In the sample there was a fair balance between genders, where 46 male (47.9%) and 50 female (52.1%) dancers completed the questionnaire, consistent with the paired nature of this dance form. Average age of respondents is 29.9 years (*SD* = 8.5, min. 19 years, max. 51 years). In terms of education, 73 people have a university degree in dance (76%), and 23 dancers have other qualifications (24%). Regarding marital status, 37 dancers are single (38.5%), 54 dancers are married or in a relationship (56.3%), and 5 dancers marked other answers (5.2%). Among the dancers 72 have no children (75%), and 24 have 1–3 children (25%).

Participants reported an average of 21.5 years of dance experience (*SD* = 8.6, min. = 6 years, max. = 43 years). Professional dance experience ranged from 3 months to 39 years, with an average of 9.1 years (*SD* = 8.8) (Table 1).

### 2.2. Measurements

The results presented in this study are part of a larger research project, which includes several questionnaires to assess the physical condition and psychosocial state of dancers. In order to ensure uniform processing, a five-point Likert scale was used in each questionnaire. The following questionnaires were used in this study.

The 14-item Perceived Stress Scale (PSS) is one of the most commonly used questionnaires to measure perceived stress, asking about feelings and thoughts that represent an individual’s perception of stress [55], adapted to Hungarian by Stauder and Konkoly Thege [56]. The original questions refer to a one-month period, but, in this research, we asked for responses going back a year. The questions express a general emotional state, such as “How often during the past year have you felt nervous and stressed?” or “How often during the past year have you been able to manage your time?” The questionnaire contains both direct and reverse items. The reliability of the PSS was excellent (Cronbach’s α = 0.86), indicating high internal consistency. The 15-item Athlete Burnout Questionnaire (ABQ) was developed by Raedeke and Smith [57] and adapted to Hungarian by Kovács et al. [58]. The questionnaire asks about the athlete’s attitude towards sport. In this research, the sport-related questions were adapted to dance; for example, the item “I am exhausted by the physical and mental demands of sport.” was modified to “I am exhausted by the physical and mental demands of dance.” The questionnaire consists of three subscales. The first subscale is reduced sense of accomplishment with five items (e.g., “I am not performing to my ability in dance.”), and the second is physical and emotional exhaustion with five items (e.g., “I feel exhausted from dancing.”). The third is sport devaluation (called “Dance devaluation” in the current study), which contains five items (e.g., “I have negative feelings about dancing.”). The reliability of all three subscales during this study was good: for the reduced sense of accomplishment subscale, α = 0.77; for the physical and emotional exhaustion subscale, α = 0.90; and for the dance devaluation subscale, α = 0.84.

The Athletic Coping Skills Inventory (ACSI-28) was authored by Smith et al. [29] to assess athletes’ coping skills. Hungarian adaptation was prepared by Jelinek [59]. The original questionnaire’s 28 questions are divided into seven subscales, of which four subscales were used in our research: Concentration with four items (e.g., “I handle unexpected situations in dance very well.”), freedom from worry with four items (e.g., “I worry quite a bit about what others think of my performance.”), confidence and achievement motivation with four items (e.g., “I feel confident that I will dance well.”), and coachability with four items (e.g., “When a coach or manager criticizes me, I become upset rather than feel helped.”). The sport-specific terms in the questionnaire were adapted for dancers. The reliability of the confidence and achievement motivation subscale was not found to be adequate (Cronbach’s α = 0.44), and no item deletion could improve the reliability; therefore, this subscale was excluded from further analyses. The reliability of the other three subscales was adequate: for the concentration subscale, α = 0.74; for the freedom from worry subscale, α = 0.78; and for the coachability subscale, α = 0.68, indicating acceptable internal consistency.

The Coaching Behavior Questionnaire (CBQ) is used to assess how athletes perceive their coach’s behavior, which is essential to maintaining concentration and an optimal mental state [60]. It has two subscales: the seven-item negative activation (e.g., “When my coach appears uptight, I don’t dance well.”) and the supportiveness subscale with eight items (e.g., “Criticism from my coach is done in a constructive manner.”). The questionnaire was adapted for Hungarian by Kovács et al. [61]. The sport-specific terms in the questionnaire were adapted for dancers. Both subscales of the questionnaire survey showed excellent reliability during this study: α = 0. 89 for the negative activation subscale and α = 0. 89 for the supportiveness subscale, indicating high internal consistency.

The Multidimensional Scale of Perceived Social Support (MSPSS) is an instrument designed to measure an individual’s perception of support. It was developed by Zimet et al. [62]. The questionnaire measures subjective social support on three subscales: family (four items), friends (three items), and significant others (three items). The Hungarian version was validated by Papp-Zipernovszky et al. [63]. In the present study, we used the family and friends subscales, with a total of ten items (e.g., “I can talk about my problems with my family.” and “My friends really try to help me.”). Both subscales have excellent reliability, α = 0.92 and α = 0.93, respectively, indicating high internal consistency.

We also asked them in the questionnaire if they had any injuries in the past year that significantly limited or prevented them from dancing for a long time. They had to choose from a list of body parts, of which they could mark more than one.

### 2.3. Data Analysis

Demographic characteristics were examined using frequency data, and continuous variables were calculated using minimum, maximum, mean, and standard deviation. The prevalence of lower limb injuries was determined from their frequency data.

After examining the demographic data (*N* = 96), descriptive statistics were calculated for the psychological scales used in this study. The skewness and kurtosis values of the psychological scales used in the analyses suggest a normal distribution, with skewness values between −0.55 and 0.61 and kurtosis values between −0.79 and 0.89, with the exception of the MSPSS scale, where family and friends subscales do not follow a normal distribution based on their skewness and kurtosis data (*S* = −2.48, *K* = 6.33 and *S* = −2.18, *K* = 4.20) (Appendix A, Table A1).

Consequently, the two groups (ankle and knee-injured and non-injured dancers) were compared along psychological subscales (excluding the MSPSS) using Multivariate Analysis of Variance (MANOVA) followed by a post hoc ANOVA. MSPPS scales were not included in MANOVA due to lack of normality and were compared using Mann–Whitney test. The homogeneity of variances was checked using Levene’s test, and based on the results, homogeneity of variances can be assumed everywhere (Appendix A, Table A2).

Since this is a cross-sectional study, this research does not attempt to identify causal relationships but rather aims to explore the associations between injuries and psychological factors.

All analyses were carried out using JASP 0.18.3. (University of Amsterdam, Amsterdam, The Netherlands).

## 3. Results

In our questionnaire, we asked whether they had an injury in the past year that significantly limited or prevented them from dancing. They could indicate injuries to more than one body part. Based on the frequency data, we found that 29 dancers had combined ankle and knee injuries in the past year, i.e., 30.2% of the total sample. We also found a higher prevalence of lower back injuries (12.5%). The prevalence of other injuries is negligible, with values of less than 10% (Table 2).

The questionnaires in the present study were used to examine the extent to which each psychosocial factor was specific to our sample and two parts of our sample, dancers with and without ankle and/or knee injuries. The corresponding mean and standard deviation results are shown in Table 1.

The comparison using the MANOVA procedure showed that ankle and knee injuries (AKIs) were associated with the psychosocial factors studied, F(1, 94) = 3.79, *p* < 0.001, Pillai’s Trace = 0.33. The results obtained by MANOVA calculation were followed by ANOVA analyses with the variables included in this study, and the results obtained are shown in Table 3.

**Figure 1:**
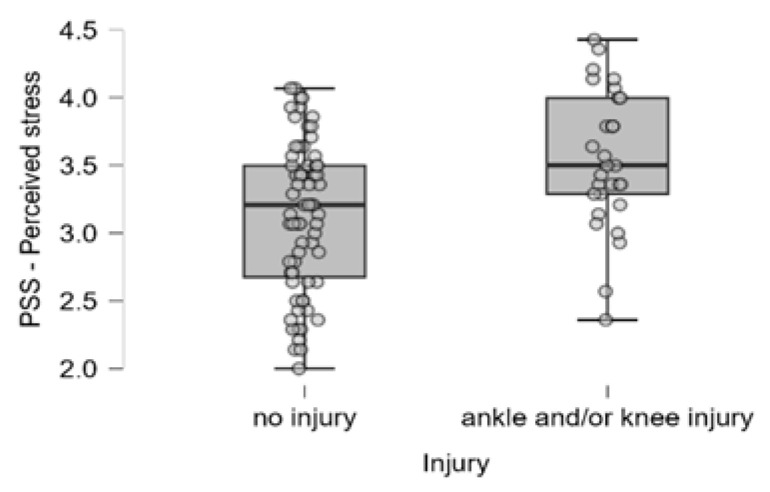
Distribution of perceived stress scores among injured and non-injured dancers. Individual dots represent raw data points to visualize variability and potential outliers. Boxes represent the interquartile range (IQR) with the median marked as a horizontal line.

**Figure 2:**
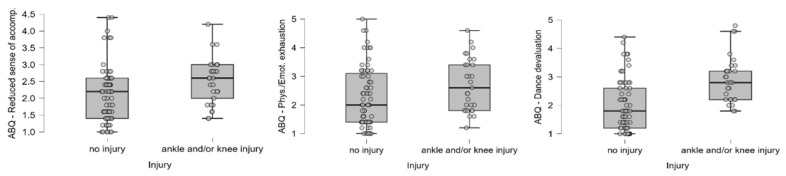
Distribution of burnout scores (reduced sense of accomplishment, emotional exhaustion, and dance devaluation) among injured and non-injured dancers. Individual dots represent raw data points to visualize variability and potential outliers. Boxes represent the interquartile range (IQR) with the median marked as a horizontal line.

Regarding psychological factors, dancers with ankle and/or knee injuries have a higher level of perceived stress (Figure 1). In terms of burnout scales, a positive correlation is seen between AKIs and a reduced sense of accomplishment and the devaluation of dance, while the difference is only marginally significant along the physical and emotional exhaustion (Figure 2).

**Figure 3:**
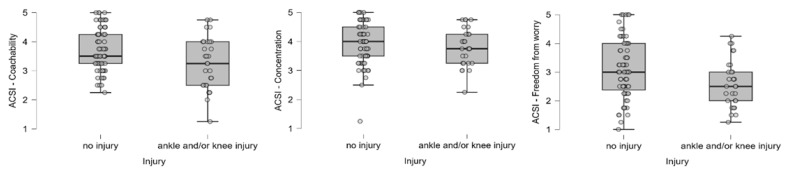
Distribution of coping skill scores (coachability, concentration, freedom from worry) among injured and non-injured dancers. Individual dots represent raw data points to visualize variability and potential outliers. Boxes represent the interquartile range (IQR) with the median marked as a horizontal line.

**Figure 4:**
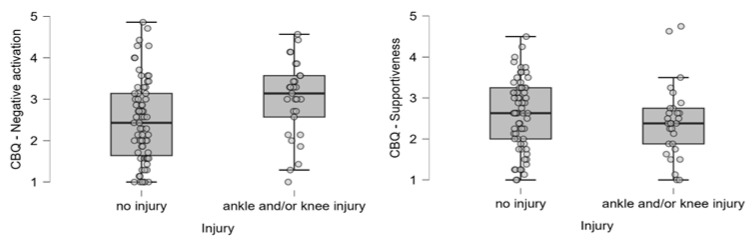
Distribution of cognitive–behavioral pattern scores (negative activation, supportiveness) among injured and non-injured dancers. Individual dots represent raw data points to visualize variability and potential outliers. Boxes represent the interquartile range (IQR) with the median marked as a horizontal line.

In examining the relationship between the scales used to measure changes in dancers’ coping skills, behavior, and attitudes towards their leader and the AKI, we found that dancers who had experienced an injury had lower levels of coachability and higher levels of worry. However, no significant differences were found in concentration (Figure 3). Furthermore, in terms of the relationship with the leader, dancers who experienced an injury had a higher prevalence of negative reactions to the leader’s behavior (Figure 4).

**Figure 5:**
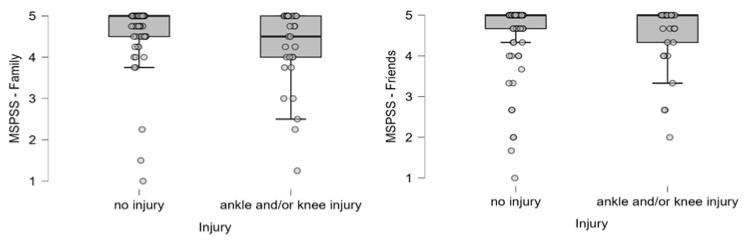
Distribution of perceived social support scores (family, friends) among injured and non-injured dancers. Individual dots represent raw data points to visualize variability and potential outliers. Boxes represent the interquartile range (IQR) with the median marked as a horizontal line.

Based on a comparison using the Mann–Whitney test, we found a significant correlation between AKIs and the level of support perceived from the family. Based on these results, the perceived social support was lower in injured dancers: *U* = 1245, *p* = 0.019, *rs* = 0.28 (AKI *M* = 4.22, *SD* = 0.98, no injury *M* = 4.63, *SD* = 0.75) (Figure 5).

We found no significant differences between younger and older dancers in terms of the occurrence of injuries and psychosocial dysfunctions during the studied period when examined by age (Appendix A, Table A3).

## 4. Discussion

In our cross-sectional research, we investigated how the mental state of Hungarian professional folk dancers is related to their injuries. Among psychological factors, we examined the relationships between perceived stress levels, the presence of burnout symptoms, the coping skills, and injuries, while among social factors, we observed the perceived social support and the relationship with the leader, in relation to injuries. All factors were examined for the year preceding this research. Given the cross-sectional nature of this study, we did not examine the causal relationship between the factors mentioned above.

With regard to the prevalence of injuries, lower limbs, specifically knee and ankle injuries, are the most common among the dancers studied, which is in line with the literature, which shows that dancers are most prone to lower limb injuries in several genres [3,9,10]. In our study, ankle and/or knee injuries occurred in 30% of the study population in the year before this study. This very high incidence rate may be related to the intensive footwork in the Hungarian folk dance, which is stressful for the lower limb joints. These findings completely confirm our first hypothesis (H1) and highlight the need to investigate the causes of injuries in order to reduce the risk of their occurrence.

Comparing the prevalence of injuries with demographic data, we found that there was no difference between dancers in terms of age. Thus, a similar prevalence can be observed among younger and less experienced dancers as among older, more experienced ones. Our findings therefore did not confirm our third hypothesis (H3). According to this result, injuries are not typically related to age. To investigate this further, it would be worth conducting research that differentiates between acute, overuse, and chronic injuries, as certain injuries become more common with age.

Regarding perceived stress, our results show that dancers who have suffered ankle and/or knee injuries in the past year have higher levels of perceived stress. This result is consistent with the stress-injury model of Williams and Andersen [20] and the correlations found by Tranaeus et al. [15] and Ivarsson et al. [23], and it confirms our second hypothesis (H2). It is a reasonable question to ask whether the high level of perceived stress contributed to the injury or whether the stress level increased due to the injury already incurred. The results show a mixed picture: on the one hand, high levels of perceived stress may indeed be a predictor of injury [15], but on the other hand, the fear of further injury after suffering an injury can also cause an increase in stress levels [16]. Due to the cross-sectional nature of this study, we are unable to establish causality. Further research would be worthwhile for this purpose. Our third hypothesis that older dancers would experience higher levels of perceived stress was not confirmed (H3).

Similarly, we found a significant relationship between ankle and/or knee injuries and some symptoms of burnout. Among those who had suffered ankle and/or knee injuries in the past year, a reduced sense of performance was observed; i.e., these dancers subjectively perceived their own performance as worse. In addition, dance as an activity also shows a devaluation in these individuals. It can be assumed that a reduced sense of performance may cause such dancers to lose interest and motivation in dance, which is one of the strongest factors in the psychological construct of burnout and may even lead to early drop-out. The presence of a significant association between injuries and these two factors of burnout (reduced sense of accomplishment and devaluation of dance) is consistent with the trend found by Madigan et al. [26] in their meta-analysis of 22 years of results and presented by Gustafsson et al. [28] and Quested and Duda [27] in their studies. Among the three characteristic dimensions of burnout, we found no significant correlation between physical and emotional exhaustion and injuries in the group studied. These findings partially confirm our second hypothesis (H2). With regard to our third hypothesis, we found that only a reduced sense of accomplishment showed a marginally significant correlation with age, while the other two dimensions did not. Thus, our third hypothesis was also only partially confirmed (H3).

Given that stress appears as one of the significant contributing factors in studies discussing burnout, the question arises whether higher stress contributes to burnout. Since our research focused on individual psychosocial factors and did not examine the interactions between them or the moderating effects of individual factors, this question could be the subject of further research. It is also worth considering conducting a qualitative study, given the complex psychological construct of burnout syndrome.

Our results demonstrate that the presence of injuries is associated with certain behavioral changes, specifically in coping skills. As mentioned in the introduction, the behavior of athletes/dancers can play a crucial role in the development of injuries and recovery after injuries, so this result is expected and also confirms partially our second hypothesis (H2). Dancers with ankle and/or knee injuries show reduced coachability; i.e., they are less likely to evaluate the leader’s instructions in a professional way, tend to misunderstand them, or are unable to accept the leader’s feedback, and, thus, the leader cannot influence their performance in an appropriate way. It has also been observed that injured dancers react more negatively to the behavior of their leader, making them more tense and even negatively affecting their performance. These two factors can also be interpreted in relation to each other, as poor interpersonal dynamics can also lead to lower coachability. Since the interaction between the individual factors was not examined in this study, it would be worthwhile to pay attention to this in further research. Our results show that dancers who suffered ankle and/or knee injuries are more concerned about their performance or potential mistakes. This result is consistent with the findings of Noh et al. [38], who, in their study examining psychosocial factors related to injuries among dancers, found that freedom from worry was significantly associated with injury frequency. Both results (decreased coachability and higher levels of worry) lead to less effective coping skills, which, according to previous research, may be a predictor of future injury. Given the cross-sectional nature of our study, we are unable to establish causal relationships in this regard. However, we can conclude that our results are consistent with those of Williams and Andersen [20] and Devantier [37], who similarly found that reduced coachability and increased levels of worry are associated with injuries. These findings confirm our second hypothesis (H2).

We found no significant correlation between injuries and concentration, which is one of the coping skills. This was a surprising result, as according to the Williams and Andersen stress-injury model [20], one manifestation of the stress response is a decrease in concentration (increased distractibility). Thus, we expected a decrease in concentration due to the higher perceived stress levels observed during this study, but this hypothesis was not confirmed (H2).

With regard to our third hypothesis, we found that concentration and freedom from worry show a significant positive correlation with age, meaning that older, more experienced dancers are better able to concentrate and do not struggle with worries to the same degree as younger dancers. We found no significant relationship between coachability and age among the coping skills. Thus, our third hypothesis was only partially confirmed with regard to coping skills (H3).

The environment plays a significant role in human life. Among athletes and dancers, it has been observed that supportive social connections, and even more so the perception of them and the relationship with the coach (as a significant part of their environment), are associated with injuries [49,52]. In our study, among the social factors, we found a significant correlation between injuries and perceived social support, as well as the relationship with the leader, which confirms our second hypothesis (H2). Dancers who have suffered ankle and/or knee injuries feel less supported by their families. Considering that the family is a significant source of emotional support, this certainly has a negative impact on their mental state. In the Williams–Andersen stress-injury model [20], one of the factors determining stress response that increases the risk of injury is social support. Thus, we can assume that reduced social support contributes to injuries through elevated stress response. Since the present study did not examine the interactions between individual psychosocial factors, this remains an open question and provides a good reason for further research. The lower level of perceived social support found in our study is in line with previous research conducted by Hardy et al. [47] with athletes and Patterson et al. [48] and Mainwaring et al. [49] with dancers.

The other significant correlation was found in the relationship with the leader. According to our results, dancers who have suffered ankle and/or knee injuries react more negatively to their leader’s behavior. This result also raises the question of whether the more negative reaction to the leader’s behavior is the cause or consequence of the injury. There are two possible explanations for this result. On the one hand, considering that the coach is a key figure in the athlete’s and dancer’s environment and social relationships, it is possible that a previously poor relationship or the dancer’s subjective perception of the coach’s behavior contributed to the injury, which is consistent with the findings of Pensgraad et al. [52]. On the other hand, it may also occur that, after an injury, dancers react more sensitively to their leader’s behavior, which is consistent with Malinauskas’ [54] findings that athletes place even greater importance on a good relationship with their coach after an injury and are therefore more sensitive to all coaching cues. As our study is cross-sectional, this question remains open, and further research is recommended to explore the causal relationships.

With regard to our third hypothesis, we found that no form of perceived social support showed a significant correlation with age, meaning that there was no difference between younger and older dancers in terms of perceived support from family and friends or attitudes toward their leader. Thus, our third hypothesis was not confirmed with regard to these factors (H3).

In summary, it is clear that the injured dancers in this study are unable to draw strength from their two main sources of support: their families in their private lives and their leaders in their professional lives.

### Limitations

In Hungary, there are approximately 130 professional folk dancers employed by professional companies. Although we were able to reach 96 of them in the present study, which represents approximately 74% of the total population, the sample size proved to be too small to allow for higher-level analyses.

A further limitation is that this research was conducted using a self-completed questionnaire, which may have bias in the results, given that dancers may have underreported both injuries and perceived psychological states.

Finally, another limitation of this study is that no distinction was made between acute, overuse, and chronic injuries. Despite this, the results provide important guidelines for practitioners, but it would be worthwhile to examine injuries in a more differentiated manner in future studies.

## 5. Conclusions

Hungarian folk dancers are prone to lower limb injuries, because of the rich footwork and relatively fast movement of this dance style, with a lot of jumping, hopping, dynamic leg swings, and lifting mostly to fairly fast music. Ankle and knee injuries are the most common, with around one third of the dancers surveyed having suffered such injuries in the year prior to this study. This research identified a correlation between injuries and certain psychosocial factors. Thus, among injured dancers, we found higher levels of perceived stress, the presence of main factors to burnout (reduced sense of accomplishment, devaluation of dance), less effective coping skills (reduced coachability, higher level of worry), more negative reactions to the leader’s behavior, and lower levels of perceived family support. All of these elements can represent a threat to a dancer’s mental health and have a negative impact on their career. Furthermore, our analysis of the correlation between injuries and psychological dysfunction and age showed that younger dancers suffer from ankle and knee injuries at a similar rate and face psychological problems at a similar level to older dancers. This means that artists need support from the very beginning of their dance careers in order to prevent and overcome these difficulties.

In conclusion, injuries are not only related to the physical condition of dancers but also to psychosocial factors. On the one hand, these factors may play a role in the development of injuries, and on the other hand, injuries may also cause psychosocial problems. Since both situations (injury and psychosocial difficulties) can lead to dancers’ decisions to end their career and leave the stage, we consider it necessary to introduce various interventions that focus on psychological assessments and support in addition to physical training and artistic work for dancers. This would be important from a preventive perspective, but it is also justified to provide psychological support to dancers after an injury in order to ensure a more effective recovery and to prevent the fear of re-injury from contributing to further injury in the future. Based on our results, a correlation between injuries and certain psychosocial factors has been proven. However, it would be desirable to examine the causal relationships. To this end, long-term studies and longitudinal research would be recommended. Since our results and previous findings suggest that certain psychosocial factors may influence injuries not only independently but also in interaction with each other, we also consider it justified to examine the moderating effects of psychosocial factors. Further investigation into the relationship between psychosocial factors and different types of injury (acute, overuse, chronic) appears to be worth considering.

Although this study was conducted among Hungarian professional folk dancers—a culturally and stylistically unique dance population—the high physical and psychological demands of the genre are comparable to those of other high-level dance art forms and can provide relevant insights for other professional dance populations.

## Figures and Tables

**Table 1:** Descriptive statistics of the sample.

**Sample Characteristics**		***n***	***%***					
Gender								
	male	46	47.9					
	female	50	52.1					
Education								
	university degree	73	76.0					
	other qualification	23	24.0					
Marital status								
	single	37	38.5					
	in a relationship/married	54	56.3					
	other	5	5.2					
Number of children								
	0	72	75.0					
	1–3	24	25.0					
**Sample Characteristics**		**Mdn**	**M**	**SD**	**Skewness**	**Kurtosis**	**Min**	**Max**
Age (in years)		27	29.9	8.5	1.0	0.1	19	51
Total dance experience (in years)		20	21.5	8.6	0.7	0.1	6	43
Professional dance experience (in years)		6.5	9.1	8.8	1.3	1.2	0.3	39

Note. *N* = 96.

**Table 2:** Prevalence of major injuries over the past year.

Body Parts	Injury Rate
Ankles	18.8%
Knees	16.7%
Ankle and knee	30.2%
Lower back	12.5%
Muscles of the lower limbs	8.3%
Neck	7.3%
Muscles of the upper limbs	5.2%
Hips	3.1%
Back	2.1%
Shoulders	1.0%

Note. *N* = 96.

**Table 3:** Examining the effect of injury on psychological factors using the MANOVA analysis.

Characteristics	No Injury		Ankle and/or Knee Injury				
	*M*	*SD*	*M*	*SD*	*F*	*p*	*η* ^2^
PSS—Perceived stress ^1^	3.13	0.56	3.54	0.51	11.78	**<0.001**	0.11
ABQ—Reduced s. of accompl. ^2^	2.11	0.83	2.52	0.68	5.51	**0.021**	0.06
ABQ—Phys./emot. exhaustion ^3^	2.30	1.06	2.71	0.93	3.21	0.077	0.03
ABQ—Dance devaluation	2.00	0.90	2.92	0.81	22.43	**<0.001**	0.19
ACSI ^4^—Coachability	3.74	0.73	3.26	0.90	7.85	**0.007**	0.08
ACSI—Concentration	3.89	0.73	3.83	0.63	0.17	0.68	0.001
ACSI—Freedom from worry	3.10	1.03	2.62	0.81	4.95	**0.029**	0.05
CBQ ^5^—Negative activation	2.49	1.01	3.01	0.92	5.66	**0.019**	0.06
CBQ—Supportiveness	2.61	0.85	2.41	0.90	1.06	0.305	0.01

Note. Data are calculated from the ANOVA analysis of variance following the MANOVA analysis. ^1^ PSS—Perceived Stress Scale. ^2^ ABQ—Athletic Burnout Questionnaire. ABQ—Reduced Sense of Accomplishment. ^3^ ABQ—Physical and Emotional Exhaustion. ^4^ ACSI—Athletic Coping Skills Inventory. ^5^ CBQ—Coaching Behaviour Questionnaire. Bold font indicates statistical significance at *p* < 0.05.

## Data Availability

The original contributions presented in this study are included in this article. Further inquiries can be directed to the corresponding author(s).

## Abbreviation

The following abbreviation is used in this manuscript:

AKIs	ankle and/or knee injuries

## Appendix A

**Table A1:** Descriptive statistics, skewness, and kurtosis values for psychological variables.

Variables	Valid	Mean	Std. Deviation	Skewness	Std. Error of Skewness	Kurtosis	Std. Error of Kurtosis
PSS—Perceived stress ^1^	96	3.25	0.57	−0.21	0.25	−0.66	0.49
ABQ Reduced s. of accompl. ^2^	96	2.23	0.81	0.61	0.25	0.13	0.49
ABQ Phys./emot. exhaustion ^3^	96	2.43	1.03	0.48	0.25	−0.72	0.49
ABQ Dance devaluation	96	2.28	0.97	0.58	0.25	−0.39	0.49
ACSI ^4^ Coachability	96	3.59	0.81	−0.17	0.25	−0.46	0.49
ACSI Concentration	96	3.87	0.70	−0.55	0.25	0.89	0.49
ACSI Freedom from worry	96	3.04	0.99	−0.29	0.25	−0.63	0.49
CBQ ^5^ Negative activation	96	2.64	1.01	0.10	0.25	−0.79	0.49
CBQ Supportiveness	96	2.55	0.87	0.18	0.25	−0.29	0.49
MSPSS ^6^ Family	96	4.51	0.84	−2.48	0.25	6.33	0.49
MSPSS Friends	96	4.51	0.88	−2.18	0.25	4.20	0.49

Note. *N* = 96. ^1^ PSS—Perceived Stress Scale. ^2^ ABQ—Athletic Burnout Questionnaire. ABQ—Reduced Sense Of Accomplishment. ^3^ ABQ—Physical and Emotional Exhaustion. ^4^ ACSI—Athletic Coping Skills Inventory. ^5^ CBQ—Coaching Behaviour Questionnaire. ^6^ MSPSS—Multidimensional Scale of Perceived Social Support.

**Table A2:** Testing homogeneity of variance using Levene’s test.

Variables	SD of Injured Dancers	SD of Non-Injured Dancers	*F*	*p*
PSS—Perceived stress ^1^	0.51	0.56	0.78	0.38
ABQ—Reduced s. of. accompl. ^2^	0.68	0.83	1.12	0.29
ABQ—Phys./emot. exhaustion ^3^	0.93	1.06	0.52	0.47
ABQ—Dance devaluation	0.81	0.90	0.94	0.33
ACSI ^4^—Coachability	0.90	0.73	2.14	0.15
ACSI—Concentration	0.63	0.72	0.63	0.43
ACSI—Freedom from worry	0.81	1.03	2.25	0.14
CBQ ^5^—Negative activation	0.92	1.01	0.99	0.32
CBQ—Supportiveness	0.90	0.85	0.32	0.57

Note. *N* = 96. ^1^ PSS—Perceived Stress Scale. ^2^ ABQ—Athletic Burnout Questionnaire. ABQ—Reduced Sense of Accomplishment. ^3^ ABQ—Physical and emotional exhaustion. ^4^ ACSI—Athletic Coping Skills Inventory. ^5^ CBQ—Coaching Behaviour Questionnaire.

**Table A3:** Correlations between age and injury occurrence and psychological variables.

Variable	*r*	*p*
Injury	0.0144	0.8892
PSS—Perceived stress ^1^	−0.0007	0.9949
ABQ ^2^—Reduced sense of accomplishment	−0.1958	**0.0559**
ABQ—Physical and emotional exhaustion	−0.0103	0.9209
ABQ—Dance devaluation	0.0005	0.996
ACSI ^3^—Coachability	−0.0573	0.5795
ACSI—Concentration	0.3189	**0.0015**
ACSI—Freedom from worry	0.2896	**0.0042**
CBQ ^4^—Negative activation	−0.0831	0.4206
CBQ—Supportiveness	−0.0467	0.6515
MSPSS ^5^—Family	−0.0115	0.9111
MSPSS—Friends	−0.156	0.1291

Note. *N* = 96. All analyses are carried out using Pearson correlation except for the MSPSS scales where Spearman correlation is used. ^1^ PSS—Perceived Stress Scale. ^2^ ABQ—Athletic Burnout Questionnaire. ^3^ ACSI—Athletic Coping Skills Inventory. ^4^ CBQ—Coaching Behaviour Questionnaire. ^5^ MSPSS—Multidimensional Scale of Perceived Social Support. Bold font indicates statistical significance at *p* < 0.05.
